# Rapid Liquid AP-MALDI MS Profiling of Lipids and Proteins from Goat and Sheep Milk for Speciation and Colostrum Analysis

**DOI:** 10.3390/proteomes8030020

**Published:** 2020-08-21

**Authors:** Cristian Piras, Carlotta Ceniti, Evita Hartmane, Nicola Costanzo, Valeria Maria Morittu, Paola Roncada, Domenico Britti, Rainer Cramer

**Affiliations:** 1 Department of Chemistry, University of Reading, Reading RG6 6DX, UK; c.piras@reading.ac.uk (C.P.); e.hartmane@pgr.reading.ac.uk (E.H.); 2 Department of Health Sciences, University “Magna Græcia” of Catanzaro, Campus Universitario “S. Venuta”, Viale Europa, I-88100 Catanzaro, Italy; ceniti@unicz.it (C.C.); costanzo.nic@unicz.it (N.C.); morittu@unicz.it (V.M.M.); roncada@unicz.it (P.R.); britti@unicz.it (D.B.)

**Keywords:** goat–sheep speciation, colostrum, protein–proteoform profiling, MALDI, mass spectrometry

## Abstract

Rapid profiling of the biomolecular components of milk can be useful for food quality assessment and for food fraud detection. Differences in commercial value and availability of milk from specific species are often the reasons for the illicit and fraudulent sale of milk whose species origin is wrongly declared. In this study, a fast, MS-based speciation method is presented to distinguish sheep from goat milk and sheep colostrum at different phases. Using liquid atmospheric pressure (AP)-matrix-assisted laser desorption/ionisation (MALDI) MS, it was possible to classify samples of goat and sheep milk with 100% accuracy in one minute of data acquisition per sample. Moreover, an accuracy of 98% was achieved in classifying pure sheep milk samples and sheep milk samples containing 10% goat milk. Evaluating colostrum quality and postnatal stages represents another possible application of this technology. Classification of sheep colostrum samples that were collected within 6 hours after parturition and 48 hours later was achieved with an accuracy of 84.4%. Our data show that substantial changes in the lipid profile can account for the accurate classification of colostrum collected at the early and late time points. This method applied to the analysis of protein orthologs of different species can, as in this case, allow unequivocal speciation analysis.

## 1. Introduction

Production and consumption of goat and sheep milk are increasing worldwide due to their nutritional and antiallergenic properties [1]. Increasing demand, differences in commercial value and the (seasonal) availability of milk originating from different species can promote the fraudulent sale of adulterated products both for direct consumption and for cheesemaking [2,3]. Therefore, accurate, fast and robust analysis is important for determining the final commercial value of such products as well as for the safety of consumers, in particular those who are prone to specific allergies [2,3,4,5].

Analyses of proteins and proteoforms for profiling purposes employing capillary electrophoresis [6], two dimensional electrophoresis [7] and HPLC [8] have previously been reported. These methods, however, are quite time consuming (at least 1 hour) due to the electrophoretic or chromatographic separation.

Techniques used for colostrum analysis and differentiation from mature milk have the same issues regarding analytical speed. Colostrum tends to be richer in proteins and lipids than milk and, in particular, is far richer in immunoglobulin levels [9,10]. Inability of newly born ruminants to ingest or to absorb sufficient amounts of good quality colostrum can result in failure of passive immunity transfer (FPT) which, in turn, leads to higher mortality and lower performance in milk production [11]. Furthermore, the eventual presence of colostrum in bulk milk can lead to numerous issues in industrial processes due to the associated changes in physical characteristics such as heat stability [12,13], negatively interfering with milk transformation processes like cheesemaking [12]. Therefore, assessing colostrum changes over time is not only important for the optimisation of milk quantity but also for the dairy industry’s food technological processes.

There are no criteria that accurately define the end of the colostrum period and the onset of main-phase lactation for any species. Immunoglobulin G (IgG) measurement has traditionally been required to establish the absence of colostrum in the domestic consumer milk supply [14]. Several techniques, such as ELISA [15], capillary electrophoresis [16] or more traditional methods like Brix refractometry [17] and the use of colostrometers [18] have been employed for IgG detection. The most widely used method for such analysis is radial immunodiffusion [10]. The wide applicability of this technique results from its simplicity and low cost despite being time consuming and affected by potential manufacturer-to-manufacturer variation [19].

In this context, the advantages of using liquid atmospheric pressure (AP)-matrix-assisted laser desorption/ionisation (MALDI) mass spectrometry (MS) allow for both the rapid accurate classification of sheep and goat milk (including mixtures) and the rapid stage classification of sheep colostrum.

## 2. Materials and Methods 

### 2.1. Sample Collection

The samples collected for this study represent a fraction of the milk collected for commercial purposes or, in the case of colostrum, for use within the farm. Sample collection was carried out in accordance with the EU Directive 2010/63/EU for animal experiments.

### 2.2. Milk Sampling for Goat/Sheep Speciation

Individual milk samples from Sarda sheep (n = 44) and from Nicastrese goats (n = 43) were manually collected from 6 different farms during the afternoon milking session. Samples were collected in sterilised tubes and kept at 4 °C for no longer than 4 h before freezing. A fraction of the sample was used for the composition analysis and the remaining part was stored at −80 °C until MALDI MS analysis, which was performed within 2 months from the sample collection date.

### 2.3. Colostrum Sampling

Sixteen Sarda sheep were enrolled in this study. During the entire experimental period the animals did not show any health problems. Colostrum samples were taken from each sheep at time 0 hours (within six hours from parturition) and at 48 hours after the first collection. Sheep were milked manually and the sampled colostrum (50 mL) was kept refrigerated (4 °C) during the transfer (<4 h) to the laboratory at the University of Catanzaro, Italy. Once received, the samples were stored at −80 °C until further analysis.

### 2.4. Conventional Milk Sample Analysis

For each milk sample, an aliquot was used to determine the amount of several milk components. Each aliquot was gently shaken and heated to 40 °C for 15 minutes. All aliquots were analysed for fat (%), protein (%), lactose (%), casein (%), urea (mg/dL), freezing point (mC), β-hydroxybutyrate (mM) and acetone (mM) using a MilkoScan FT+ analyser (Foss Electric, Hillerød, Denmark).

### 2.5. Sample Preparation for MS Analysis

The extraction of lipids was performed according to the method reported by Calvano et al. [20]. Briefly, the extraction was carried out as follows: a volume of 187 µL of CHCl_3_:MeOH (1:2; v:v) was added to 50 μL of milk/colostrum. Subsequently, water (62 µL) and chloroform (62 µL) were added. The resulting solution was vortexed and centrifuged at 11,000× *g*. The lower organic phase was transferred to a clean vial and used as analyte solution.

For combined lipid and peptide/protein analysis, aliquots (50 µL) of milk (sheep or goat), milk mixtures (100% sheep, 95% sheep/5% goat, 90% sheep/10% goat) or colostrum were precipitated with 250 µL of 5% (w:v) trichloroacetic acid. The pellet was re-suspended in 200 µL of water:acetonitrile:isopropanol (1:1:1; v:v:v) solution and used as the analyte solution.

For solid-state MALDI MS analysis, 1 µL of analyte solution was mixed with 1 µL of 4-chloro-α-cyano-cinnamic acid (CClCA) matrix solution (5 mg/mL in MeOH, 0.1% TFA (v:v)) on the MALDI sample plate.

For liquid AP-MALDI MS analysis, α-cyano-4-hydroxycinnamic acid (CHCA) was used as the MALDI matrix chromophore. Briefly, 30 mg/mL CHCA was dissolved in acetonitrile/water (70:30; v:v) by 2-min sonication. This solution was subsequently diluted 10:7 (v:v) with ethylene glycol forming the liquid support matrix (LSM) solution. Immediately before MS analysis, 0.7 µL of analyte solution was mixed with 0.7 µL of LSM solution directly on the MALDI sample plate.

### 2.6. MS Analysis

The lipid extracts were analysed by solid-state MALDI MS on a MALDI Quadrupole Time-of-Flight (Q-TOF) Premier mass spectrometer (Waters Corporation, Wilmslow, UK) over an *m/z* range of 500–2000. External mass calibration was obtained using a 600–1200 polyethylene glycol (PEG) mixture.

The combined lipid and protein MS analyses were performed by liquid AP-MALDI MS [21,22,23] on a Synapt G2-Si High Definition Mass Spectrometry (HDMS) mass spectrometer (Waters Corporation) equipped with an in-house developed AP-MALDI source, using a Waters research enabled software (WREnS)-controlled XY stage (Zaber Technologies Inc., Vancouver, Canada). A detailed source description has been reported previously [24].

### 2.7. MS Profile Data Analysis

Raw datasets were analysed with Abstract Model Builder (AMX; version 1.0.1563.0; Waters). AMX was used to create linear discriminant analysis (LDA) models. The validation of each model created was assessed by the software’s built-in “leave 20% out”. For both lipids and the combined lipid/peptide/protein analysis, models were created using the entire *m/z* range of 500–2000.

For obtaining average masses and charge states of highly charged ion species, MS spectra and charge state distributions were further analysed with UniDec software (version 1.0.10, University of Oxford, Oxford, UK) [25].

### 2.8. MS/MS Analysis

For protein identification by MS/MS, 40 V collision energy was applied to the transfer cell and the LM (low-mass) resolution of the quadrupole was set to 6. Data were acquired in the *m/z* range of 100–2000 with ion mobility. The raw data file was analysed with Mascot Distiller (Version 2.7.1.0, 64-bit; Matrix Science, London, England) for automated peak picking. The obtained peak list consisted of the monoisotopic masses of the singly charged equivalents of the fragment ions detected. This peak list was exported as a text file and searched using the MS-Tag function of ProteinProspector (v 6.2.1; http://prospector.ucsf.edu/) against the UniProtKB.2017.11.01 database restricted to *Ovis aries* as species. The search parameters were set as follows: no enzyme, monoisotopic masses, 5 Da for precursor ion tolerance and 0.2 Da for fragment ions, using the default setting for variable modifications.

## 3. Results

For the goat and sheep milk samples, the MilkoScan data of the analysis of the various milk components are shown in Table 1.

**Figure 1:**
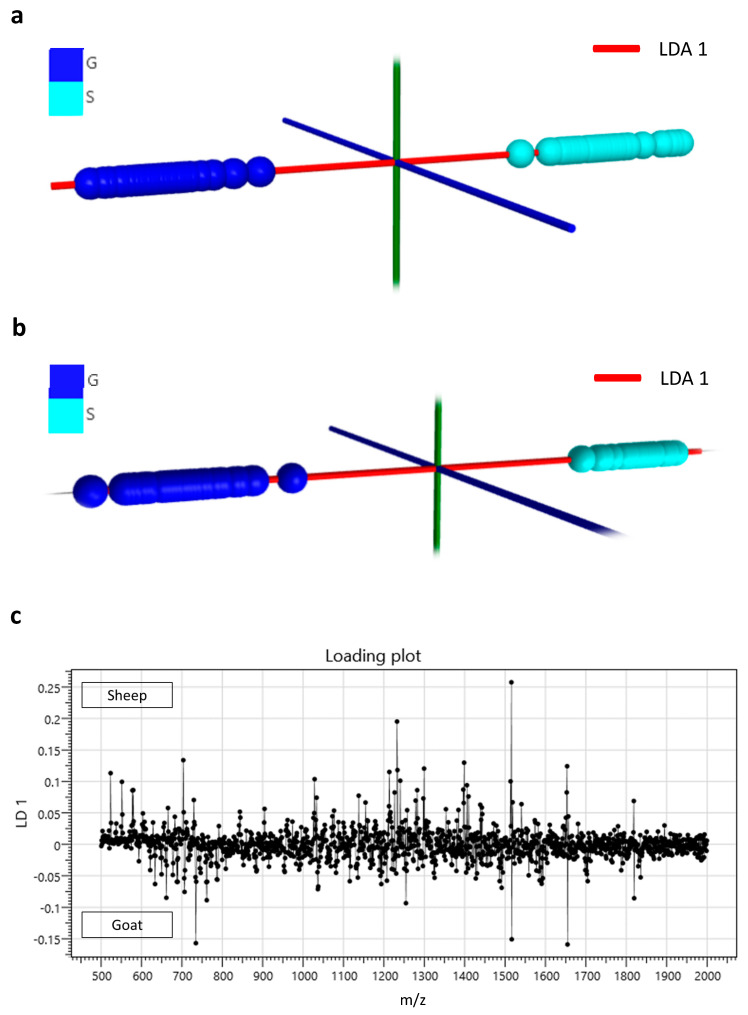
(**a**) Linear discriminant analysis plot obtained from solid-state matrix-assisted laser desorption/ionisation (MALDI) MS profiling data of the lipids extracted from goat (G) and sheep (S) milk samples. (**b**) Linear discriminant analysis plot obtained from liquid atmospheric pressure (AP)-MALDI MS profiling data of both lipids and proteins from goat (G) and sheep (S) milk samples. (**c**) Loading plot of the linear discriminant analysis of the data obtained by liquid AP-MALDI for the classification of goat and sheep milk samples shown in (**b**).

Profiling of milk lipids for speciation was initially performed by conventional (solid-state) MALDI MS as described in the methods section. Using solid-state MALDI MS for the analysis of all goat and sheep samples, it was possible to achieve a species classification accuracy of 96.6%. Figure 1a demonstrates the LDA plot obtained from this classification analysis. Full profiling of both lipids and proteins using liquid AP-MALDI MS led to faster and more accurate classification of all goat and sheep milk samples than the solid-state MALDI MS analysis, which lacked significant signal intensity from proteinaceous ions. The LDA classification data shown in Figure 1b were obtained using liquid AP-MALDI MS acquisitions of 1 minute per sample, resulting in a classification accuracy of 100%. The liquid AP-MALDI MS profile ions most relevant to this high classification accuracy are in the ion bins at *m/z* 1513.5 and 1516.5 of the loading plot for this LDA analysis (see Figure 1c).

**Figure 2:**
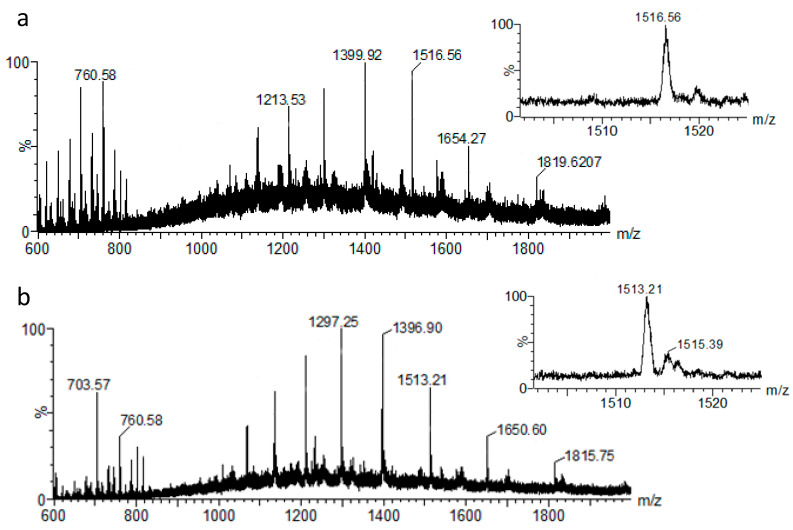
Representative liquid AP-MALDI MS spectra of goat (**a**) and sheep (**b**) milk over an *m/z* range of 600–2000. The insets display the enlarged spectral region for the putatively assigned proteoforms of β-lactoglobulin.

Figure 2 displays representative spectra that were obtained from the liquid AP-MALDI MS analysis of goat and sheep milk. The *m/z* 1500–1530 regions in these spectra (insets in Figure 2) clearly show the MS ion signals for the above loading plot ion bins. These signals can be assigned to the [M + 12H]^12+^ ions of the charge state series of molecules with an average mass of 18,198.7 Da and 18,158.5 Da, respectively, as calculated by UniDec software. The mass difference between the ions of these two charge state series is ~40 Da.

MS/MS analysis of the peak at *m/z* 1513.21 in the MS profile of the sheep milk analysis (see Figure 2b) resulted in a list of 135 fragment ion signals after peak picking using the Distiller software. The monoisotopic masses of this fragment ion peak list were searched against a UniProtKB protein database restricted to *Ovis aries*. The results from this search identified β-lactoglobulin as the most probable protein match with the least unmatched fragment ions. Although the identification of β-lactoglobulin was achieved using a 5-Da precursor ion mass tolerance, it included the default variable modifications (mainly methionine oxidation and N-terminal modifications). All matches for β-lactoglobulin suggested that it had been modified on one of its methionines. Consequently, a search at 5-Da mass tolerance but without the default variable modifications eliminated β-lactoglobulin from the protein match results and the top match had a much lower score and less fragment ions matching. On the other hand, searches with a precursor ion mass tolerance greater than the mass gain of methionine oxidation (plus 5 Da) identified β-lactoglobulin as the top match. Thus, to allow for other potential modifications these searches should generally be conducted with a higher precursor ion mass tolerance to allow for *a priori* unknown modifications. The exact mass tolerance to be applied can obviously be a matter of debate.

**Figure 3:**
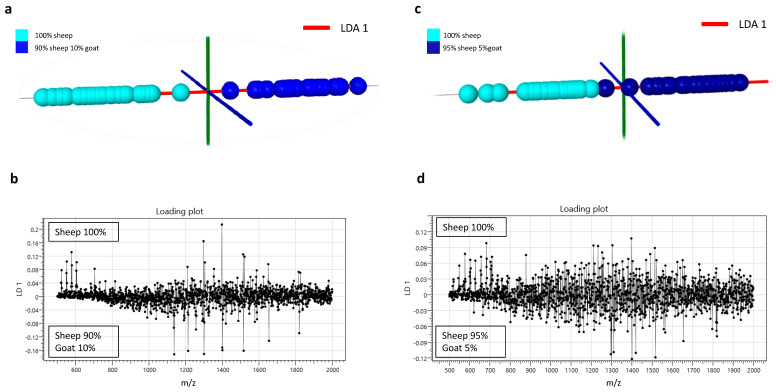
(**a**) Linear discriminant analysis plot and (**b**) its loading plot for the data obtained by liquid AP-MALDI MS profiling for the classification of sheep milk samples and sheep milk samples with 10% goat milk. (**c**) Linear discriminant analysis plot and (**d**) its loading plot for the data obtained by liquid AP-MALDI MS profiling for the classification of sheep milk samples and sheep milk samples with 5% goat milk.

Using liquid AP-MALDI MS profiling it was also possible to detect goat milk at low amounts in sheep milk. Pure sheep milk samples and sheep milk samples with 10% goat milk were classified with an accuracy of 98% (Figure 3a,b). Lowering the level to 5% goat milk in sheep milk still resulted in a classification accuracy of 72% (Figure 3c,d).

**Figure 4:**
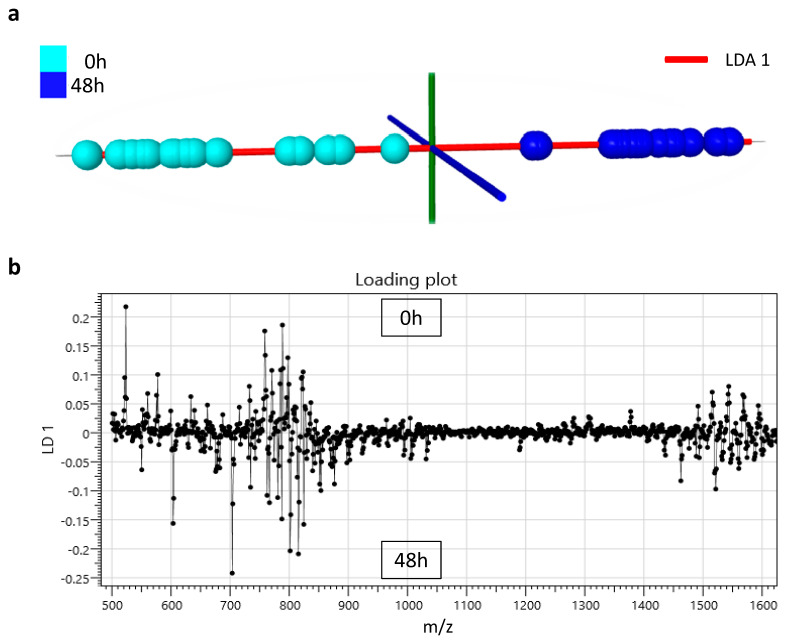
(**a**) Linear discriminant analysis plot obtained from conventional (solid-state) MALDI MS profiling data of Sarda sheep colostrum samples collected at time 0 h (light blue) and 48 h (grey) later. (**b**) Loading plot of the linear discriminant analysis of the data obtained by conventional (solid-state) MALDI for the classification of Sarda sheep colostrum samples collected at time 0 h and 48 h later.

For the colostrum study, milk samples from 16 Sarda sheep were collected for each time point and analysed by conventional (solid-state) MALDI and liquid AP-MALDI MS. As can be seen in Figure 4, just using the lipid profiles obtained from conventional MALDI MS and LDA, it was possible to distinguish between the colostrum collected within 6 h after parturition and that collected 48 h later from the same animals. The classification accuracy for this analysis was 84.4%. The LDA loading plot in Figure 4b highlights the ions responsible for this classification while Table 2 lists the corresponding possible identifications for these ions. Listed ion signal peaks were chosen according to their relevance (as determined by LDA) for distinguishing between colostrum collected within 6 h of birth and 48 h later.

When the analysis was performed using the entire spectral profiling, including the multiply charged proteinaceous ions, the classification accuracy was not significantly improved (data not shown).

## 4. Discussion

Adulteration and quality assessment of milk require a rapid, affordable, sensitive and reliable method. Methods such as near-infrared (NIR), nuclear magnetic resonance (NMR) and electrophoresis could be used and, to date, the European Union reference method to detect cow milk in other species’ milk is based on isoelectric focusing of γ-caseins [26]. These methods are time consuming and focused on the detection of cow milk in milk of other species.

The molecular composition of milk is influenced by several factors, including but not limited to animal species, breed, genotype, diet, and stage of lactation [27]. As expected, sheep milk had greater amounts of fat, protein, casein and lactose in comparison to goat milk (Table 1) [28].

MALDI MS profiling of milk using hybrid Q-TOF instrumentation allows the simultaneous detection of both lipids and peptides/proteins from the same mass spectrum. In particular, liquid AP-MALDI MS is well suited for the detection of peptides and proteins as it produces multiply charged peptide and protein ions [21]. All this can be achieved with a simple one-pot sample preparation [29]. Using liquid MALDI with its liquid sample droplets also avoids the typical search for sample “sweet spots” to achieve optimal desorption/ionisation and provides superior ion yield stability [30,31].

Liquid AP-MALDI MS profiling analysis applied to goat and sheep milk resulted in a species classification accuracy of 100% using samples from 87 individual animals. As can be seen from the loading plot presented in Figure 3, the ion signals mainly responsible for this classification can be found in the *m/z* 1000–2000 region, where typically most of the ion signals for multiply charged proteins are detected.

Among the differentially recorded proteins of the loading plot in Figure 3, the most relevant ions for this classification were detected at *m/z* 1513.21 and 1516.56. These ions can be assigned to [M + 12H]^12+^ ion species with an average mass of 18,158.5 Da and 18,198.7 Da, respectively. From the MS/MS analysis of the [M + 12H]^12+^ ions of the sheep milk sample, β-lactoglobulin was identified. According to the UniProtKB database entries of β-lactoglobulin for sheep (P67976) and goat (P02756), the mass difference between the mature forms of these two protein orthologs is approximately 40 Da, which is the same as the mass difference between the detected ion signals in the sheep and goat MS profiles. Thus, it can be concluded that β-lactoglobulin was detected in the MS profiles of both species and can be used as a species–specific ion signature for unequivocal classification. Previous analysis of cow milk using the same liquid AP-MALDI MS methodology also demonstrated the detection of β-lactoglobulin at 18.3 kDa [29]. As is visible in the LDA loading plots of Figure 1c and Figure 3b,d, the differential detection of β-lactoglobulin proteoforms (e.g., *m/z* bins at 1513 and 1516, respectively) allowed the highly successful detection of 10% goat milk in sheep milk. This result is particularly relevant considering the relatively low phylogenetic distance of these two species and the preparative and analytical speed of the method. However, to obtain a classification accuracy as high as 98% for the sheep milk adulteration with 10% goat milk, it was necessary to use the entire MS profile. Using just the peaks at *m/z* 1513.21 and 1516.56 only led to a classification accuracy of 81.5%, indicating that signal-to-noise levels are already substantially lower at the 10% adulteration level and that a panel of peaks or an entire profile can improve the robustness of the classification.

The MS analysis time for the above samples was just one minute per sample and can be further reduced if the data acquisition rate is optimised [32], e.g., by using a laser with a higher pulse repetition rate, a faster sample holder stage, and by limiting the number of scans to the minimum for detecting sufficient ion signal intensities of the analytically important peaks.

Although MALDI MS analysis on axial, high-vacuum TOF instruments has been increasingly used to study milk [33], the use of Q-TOF instrumentation, in particular in combination with liquid AP-MALDI, is relatively new and offers the advantage to detect small molecules such as lipids, as well as larger molecules, such as peptides and proteins, without the large amount of matrix background ion signal as usually experienced with linear axial MALDI-TOF MS measurements [34]. Similarly, the characterisation of colostrum is still rare with virtually no reported MALDI MS profiling analyses and generally limited to only a few studies.

Li et al. [35] recently analysed lipids in donkey milk during different lactation periods using a quantitative MS-based lipidomic approach. Overall, 335 different lipids belonging to 13 subclasses were identified in both donkey colostrum and milk, of which 60 lipids were found to be up-regulated and 43 down-regulated as a result of the transition from colostrum to milk.

Similar studies performed on cow milk highlighted strong differences in ceramides between colostrum and mature milk. These changes mainly represent a decrease in saturated fatty acids (SFA) and long chain fatty acids content in mature milk [36]. Similarly, bovine colostrum sampled at day 1 after delivery had a higher content of phospholipids. Phosphatidylethanolamine and sphingomyelin contents were lower and higher, respectively, in the first 5 days after calving in comparison to five months [37].

Changes in fatty acid (FA) composition are also reported in yak colostrum. Yak colostrum produced at the earlier stages had lower SFA and higher monounsaturated FA (MUFA) and poly unsaturated FA (PUFA) levels than the colostrum collected in subsequent days [38].

In the current study, 19 easily detectable molecules were found to be potential lipid markers for classifying the lactation stages of sheep. Individual colostrum samples collected within 6 hours after delivery and 48 hours later were classified with 84.4% accuracy.

Table 2 lists the ion signals mostly contributing to the stage classification of the colostrum samples. Lipids assignments were made by using the LIPID MAPS database (http://www.lipidmaps.org/) and published data [20]. The ions at *m/z* 523.47 and 703.57 strongly contributed to the classification of samples at 0 and 48 hours.

Our results based on the LDA loading plot identified ceramide (d32:3) as one of the most relevant lipid markers for discriminating the sheep lactation stages (Table 2 and Figure 4b. These results are in agreement with previously reported qualitative and quantitative changes in the lipid and protein profiles during early lactation of dairy animals.

## 5. Conclusions

This work demonstrates the efficacy of liquid AP-MALDI MS profiling for the fast classification of milk from different species and of mixtures through the recording of the milk’s (lipid/)protein MS profiles as well as the detected orthologs of single proteins within these profiles. This profiling method has the potential to be applied in several areas of the food and zootechnical industry, from the detection of adulterations to food quality and nutritional assessment.

MALDI MS profiling can also be applied for the classification of colostrum and mature milk. To date, the officially recognised methods for distinguishing colostrum from transitional milk and for assessing the quality of colostrum are based on the estimation of immunoglobulins using methods such as Brix refractometry and ELISA [17,39]. In the present study, for the first time MALDI MS lipid profiling was used for distinguishing colostrum collected in the early hours from the colostrum collected in the following hours. Overall, simple milk/colostrum sample preparation and rapid MALDI MS profiling on high-performing MALDI hybrid mass analysers significantly add to the MS-based detection capabilities of putative markers for species and lactation classification.

## Figures and Tables

**Table 1:** Composition data for goat and sheep milk.

Samples	Data Description	Fat (%)	Crude Protein (%)	Lactose (%)	Casein (%)	Cryoscopy (mC)	Urea (mg/dL)	True Protein (%)	Acetone (mM)	β-hydroxy-butyrate (mM)
Goat	Mean	5.49	3.97	4.66	3.05	540	52.75	3.68	0.06	0.02
(n = 43)	SD *	1.43	0.53	0.21	0.44	9	13.77	0.52	0.08	0.03
Sheep	Mean	6.6	5.35	4.71	4.03	564	40.67	4.97	0.27	0.15
(n = 44)	SD *	0.98	0.6	0.4	0.46	20	9.97	0.59	0.26	0.17

* SD, standard deviation.

**Table 2:** Putative lipid assignments of ion signal peaks measured by liquid AP-MALDI MS.

Ion Bin(*m/z*)	Peak *m/z* Values (Monoisotopic)	Theoretical *m/z* Values (Monoisotopic)	Putative Lipid Assignment
523.5	523.47	523.48	[Cer(d32:3) + NH_4_]^+^
675.5	675.51	675.53	[SM(D16:1/16:0) + H]^+^
703.5	703.57	703.57	[SM(34:1;O2) + H]^+^
706.5	706.55	706.54	[PC(30:0) + H]^+^
734.5	734.57	734.57	[PE(34:4) + Na]^+^
787.5	787.63	787.63	[SM(40:1;O2) + H]^+^
788.5	788.62	788.62	[PC(36:1) + H]^+^
800.5	801.68	801.67	[SM(d41:1) + H]^+^
815.5	815.69	815.70	[SM(42:1)+ H]^+^

Cer, ceramide; SM, sphingomyelin; PC, phosphatidylcholine; PE, phosphatidylethanolamine; PI, phosphatidylinositol.
